# Eliminating hepatitis C virus as a public health threat among HIV‐positive men who have sex with men: a multi‐modelling approach to understand differences in sexual risk behaviour

**DOI:** 10.1002/jia2.25059

**Published:** 2018-01-09

**Authors:** Nick Scott, Mark Stoové, David P Wilson, Olivia Keiser, Carol El‐Hayek, Joseph Doyle, Margaret Hellard

**Affiliations:** ^1^ Disease Elimination Program Burnet Institute Melbourne Vic. Australia; ^2^ Department of Epidemiology and Preventive Medicine Monash University Clayton Vic. Australia; ^3^ Institute of Social and Preventive Medicine University of Bern Bern Switzerland; ^4^ Department of Infectious Diseases The Alfred and Monash University Melbourne Vic. Australia

**Keywords:** agent‐based model, coinfection, elimination, hepatitis C virus, HIV, men who have sex with men, multi‐modelling

## Abstract

**Introduction:**

Outbreaks of hepatitis C virus (HCV) infections among HIV‐positive men who have sex with men (MSM) have been observed globally. Using a multi‐modelling approach we estimate the time and number of direct‐acting antiviral treatment courses required to achieve an 80% reduction in HCV prevalence among HIV‐positive MSM in the state of Victoria, Australia.

**Methods:**

Three models of HCV transmission, testing and treatment among MSM were compared: a dynamic compartmental model; an agent‐based model (ABM) parametrized to local surveillance and behavioural data (“ABM1”); and an ABM with a more heterogeneous population (“ABM2”) to determine the influence of extreme variations in sexual risk behaviour.

**Results:**

Among approximately 5000 diagnosed HIV‐positive MSM in Victoria, 10% are co‐infected with HCV. ABM1 estimated that an 80% reduction in HCV prevalence could be achieved in 122 (inter‐quartile range (IQR) 112 to 133) weeks with 523 (IQR 479 to 553) treatments if the average time from HCV diagnosis to treatment was six months. This was reduced to 77 (IQR 69 to 81) weeks if the average time between HCV diagnosis and treatment commencement was decreased to 16 weeks. Estimates were consistent across modelling approaches; however ABM2 produced fewer incident HCV cases, suggesting that treatment‐as‐prevention may be more effective in behaviourally heterogeneous populations.

**Conclusions:**

Major reductions in HCV prevalence can be achieved among HIV‐positive MSM within two years through routine HCV monitoring and prompt treatment as a part of HIV care. Compartmental models constructed with limited behavioural data are likely to produce conservative estimates compared to models of the same setting with more complex parametrizations.

## 1 Introduction

Hepatitis C virus (HCV) infection is a leading cause of death among HIV‐positive individuals 1, 2. People living with HIV have an increased risk of acquiring HCV, in particular through sexual contact 3. This is especially the case among HIV‐positive men who have sex with men (MSM) who report engaging in high‐risk sexual behaviour, sometimes but not always in association with injecting drug use 4, 5. Outbreaks of HCV infections among MSM have been described in Europe 4, 5, 6, 7, the United States 8, and Australia 9, 10. Once infected, HIV/HCV co‐infected individuals have a reduced chance of spontaneous clearance 11, 12, a more rapid progression to HCV‐related liver disease and increased risk for cirrhosis and liver cancer 13 compared to people without HIV.

The recent availability of interferon‐free direct‐acting antiviral (DAA) HCV treatment regimens means that HCV elimination is now being considered globally 14, with the World Health Organization announcing 2030 country‐level targets of reducing new chronic HCV infections by 80% and HCV‐related mortality by 65% 15. Achieving elimination will require targeted interventions among key populations, and in particular specific programmes are needed to improve the currently poor cascade of care 16. HCV testing and treatment scale‐up among HIV‐positive MSM is highly achievable with minimal extra delivery costs given their likely engagement with the healthcare system for their HIV care. Recent global guidelines recommending commencement of HIV treatment regardless of CD4 count 17 will also enhance the potential reach of DAA HCV treatments and the feasibility of HCV elimination efforts in co‐infected populations.

Mathematical models are a useful tool for estimating the feasibility and resources required to achieve HCV elimination 18, 19. Most modelling studies are restricted by data limitations to the use of compartmental models, which assume either homogeneous populations and risk behaviours or at best crudely divide populations into a small number of discrete categories 18. In reality, the risk of HIV‐positive MSM sexually acquiring or transmitting HCV is complex and related to HCV testing frequency, consistency of condom use, frequency of sexual partnerships, duration/concurrency of partnerships and many other individual characteristics that are generally either ignored or taken as average values in compartmental models. In contrast, agent‐based models (ABMs) are a type of model that can take account of individual‐level characteristics using a set of autonomous “agents” with unique characteristics to represent a population.

ABMs offer a powerful method for describing complex human behaviour, as each agent makes decisions about how to interact with other agents and their environment based on their individual characteristics. When many agents are combined and simulated together, large scale epidemiological patterns emerge from a multitude of individual behaviours and stochastic interactions. ABMs are therefore able to answer questions related to extreme between‐individual variations in risk, and can inform whether these effects warrant consideration in policy‐making.

In Victoria, Australia, a unique opportunity exists to parametrize an ABM of HCV transmission to the population of HIV‐positive MSM, in which approximately 10% are co‐infected with HCV 20. The Victorian Primary Care Network on Sentinel Surveillance (VPCNSS) 21 involves automated data draws of clinical and laboratory testing data from primary care, sexual health and laboratory service networks around the state and collects linked risk behaviour data from MSM testing at high caseload clinics. VPCNSS is capable of linking clinical and laboratory data on HCV testing to behavioural data. With this data the distribution of risk (e.g. number of partners and consistency of condom use) and protective (e.g. testing frequency) behaviours can be defined and allocated to agents in the model.

In this paper, we compare three model estimates for the time and treatment requirements to achieve an 80% reduction in HCV prevalence among HIV‐positive MSM in Victoria: a simple compartmental model (reflecting what might be estimated if only limited data were available); an ABM parametrized by VPCNSS data (reflecting a best estimate using a more complex parametrization); and an ABM with a more heterogeneous population (to test how the existence of very high‐risk individuals and behaviours not captured by surveillance systems might impact model estimates). Although the baseline parameters are aligned with Victorian epidemiological data, the model will be publically available online (https://www.openabm.org/model/5234) with population characteristics and treatment access easily modifiable to other settings globally.

## 2 Methods

### 2.1 Setting

Victoria has the second largest number of people living with HIV in Australia, with the majority living in metropolitan Melbourne; in 2013 there were estimated to be approximately 5000 diagnosed HIV‐positive MSM in Victoria 22. Total HCV notifications in Victoria have been relatively stable for the period 2011 to 2014 at between 2200 and 2350 new HCV diagnoses per year (38 to 41 per 100,000 population) 23; however a recent increase has been seen in notifications of HCV in HIV‐positive MSM, predominately driven by sexual transmission 9. Overall around 2% of Victorian HCV notifications are estimated to occur among HIV‐positive MSM, predominately through sexual exposure 9. This would represent notification rates of HCV in this population of approximately 45 cases per year (1.8 per 100 person‐years at risk 20). In addition, around 18% of HIV‐positive MSM report injecting drug use 24. The incidence of HCV through injecting drug use has been estimated previously at 11.9% per annum in Australia 25, and hence approximately 48 additional cases per year could be expected among HIV‐positive MSM through this exposure (assuming a 50% HCV prevalence among people who inject drugs 26, and therefore that 9% of the 4500 HCV‐uninfected are at risk).

A lack of behavioural data prevented the inclusion of HIV‐undiagnosed MSM in our analysis, and henceforward HIV‐positive MSM refers to only those who have been diagnosed of their HIV (in Australia an estimated 88% of people living with HIV have been diagnosed 23).

### 2.2 HCV transmission

In this study, we focussed on the sexual transmission of HCV among HIV‐positive MSM. This was both for the purposes of understanding the impact of assumptions about sexual networks and owing to a lack of detailed epidemiological and behavioural data on other transmission mechanisms within this population. However, we accounted for HCV incidence through other modes using an “HCV import” feature in each model, which allowed people to become infected from external sources and through other mechanisms. Imported infections were used as a method of accounting for transmission through injecting drug use primarily, but also transmissions through partnerships between HIV‐positive MSM and HIV‐negative MSM (which contribute substantially lower risk of HCV transmission because of lower HCV prevalence compared to HIV‐positive MSM), through partnerships with HIV‐undiagnosed MSM, or through riskier than normal sex when travelling. The imported infection feature is designed to add flexibility and has been used in this context elsewhere 27, 28.

### 2.3 Compartmental model description

**Figure 1:**
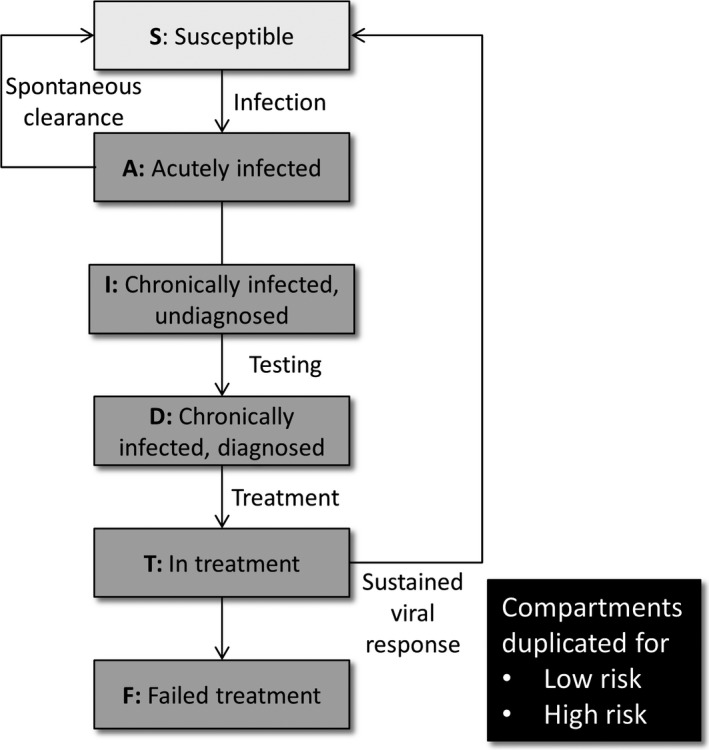
Compartmental model schematic.

We used a dynamic compartmental model to approximate HCV transmission, diagnosis and treatment among HIV‐positive MSM as shown in Figure 1. The population was categorized as either: susceptible (i.e. not HCV‐infected), acutely HCV‐infected, chronically HCV‐infected and not diagnosed, chronically HCV‐infected and diagnosed, in treatment for HCV, or failed treatment for HCV. Each compartment was stratified by high and low levels of risk, with high‐risk individuals defined as MSM who had more than 10 sexual partners, participated in group sex or used recreational drugs during sex in the past six months 29. High‐risk MSM had their risk of acquiring or transmitting HCV increased by a factor of two compared to low‐risk individuals. A detailed model description is in the supplementary material, and parameters and their sources are in Table 1.

**Table 1:** Compartmental model parameters

Description	Value	Source
Population size	5000	Unpublished Victorian surveillance data 22.
HCV prevalence	10%	Unpublished Victorian surveillance data 20.
Proportion of HIV‐positive MSM who spontaneously clear HCV infection	15%	30
Duration of acute stage	12 weeks	31
Average treatment length	17 weeks	12 weeks for genotype 1 and 2, 24 weeks for genotype 3 32, 33, 34, 35; assuming the Australian average genotype distribution (62% genotype 1 and 2, 38% genotype 3 36)
Treatment success rate	95%	For genotype 1 32, 33, 34, 37; assumed equally efficacious across genotypes.
Force of infection	–	Calibration parameter
Relative risk of infection for high‐risk group	2	Assumed
Proportion at high risk	69%	Defined according to STIGMA guidelines 29. From the Gay Community Periodic Survey 69% of HIV‐positive MSM fit the high‐risk criteria 24.
Importation of infection	3.1% per annum for high‐risk	Assumes that the majority of imported infections are through injecting drug use. Using an estimated 11.9% probability of infection per year for people who inject drugs 25, and assuming that the 18% of HIV‐positive MSM who inject drugs 24 are among the high‐risk group, then the annual probability is (18% × 5000 HIV‐positive MSM who inject drugs)/(69% × 5000 HIV‐positive MSM at high risk) × (11.9% probability per year of becoming infected)
Reduction in HCV transmission risk once HCV‐diagnosed	45%	Gay Community Periodic Survey 24: 80% disclosed STI status to any sex partner, 45% told all of their sex partners.
Testing frequency, prior to treatment scale up (average times per week)	Once per year	Testing frequency coinciding with annual HIV check‐ups. Data does not suggest this differs by risk status.
Average time from diagnosis to commencing HCV treatment, prior to treatment scale up (weeks)	200 weeks	Assumed
Testing frequency, post treatment scale up	Once per year	Assumed testing frequency to continue as part of HIV care.
Average time from diagnosis to commencing treatment, post treatment scale up	26 weeks	Assumed

The population and behaviours of HIV‐positive MSM in Victoria, before and after hypothetical treatment scale‐up programmes are implemented. MSM, men who have sex with men; HCV, hepatitis C virus.

Both before and after treatment scale‐up HIV‐positive MSM were assumed to have HCV tests as part of their HIV care. To model treatment scale‐up, the average time from diagnosis to commencing treatment was decreased from approximately four years to six months. The model was run for three years post treatment scale‐up and the number of people infected with HCV, HCV incidence and number of treatments initiated was recorded over time.

### 2.4 ABM 1 description

An ABM was constructed in NetLogo 38 and run through R using the RNetLogo package 39. It was built by modifying the existing AIDS model 40 to simulate HCV rather than HIV transmission dynamics, include different types of sexual partnerships (described below) and to reflect the characteristics of HIV‐positive MSM in Victoria. The complete model is available from the online repository https://www.openabm.org/model/5234, and a detailed model description is in the supplementary material. Parameters and their sources are in Table 2.

**Table 2:** Agent population characteristics and treatment scale‐up implementations

Agent property	Distribution	Source
Agent‐based model 1: best representation of Victorian HIV‐positive MSM population		
Proportion with regular partners	30%	Gay Community Periodic Survey 24: 30% had a current regular partner
Relationship lengths with regular partners (weeks)	Gamma (mean = 4.5 years, variance = 36 years)	Data from Van de Ven et al. 41: average time in relationship was a median = 2 years, mean = 4.5 years (SD of mean = 6.2 years)
Condom use probability with regular partners	Beta (mean = 19%, variance = 5%)	Gay Community Periodic Survey 24 for mean; Authors' opinion for variance
Condom use probability with casual partners	Beta (mean = 42%, variance = 5%)	Gay Community Periodic Survey 24 for mean; Authors' opinion for variance
Percent who have casual partners	56%	Gay Community Periodic Survey 24: 56% had a *current* casual partner. Likely to be lower bound.
Number of casual partners per year (for those who have them)	Gamma (mean = 13, variance = 46)	VPCNSS: 63% of HIV‐positive MSM had more than five casual partners in past six months (extrapolated to 10 in past 12 months); 15% had more than 10 partners in six months (or 20 in 12 months). These quantiles were used to back‐calculate gamma distribution parameters 42
Percent of casual sex with casual partners (i.e. fuck buddy as opposed to “random” partners)	95%	Authors' opinion; sensitivity analysis
Frequency of having casual sex (times per year)	Gamma (mean = 17 × number of casual partners, variance = 10)	From Rawstorne et al. 43: the 100 HIV‐positive MSM who reported having unprotected anal intercourse with other HIV‐positive MSM reported a total of 2111 acts in the previous six months. Scaling this up to account for the 42% use of condoms (see above) gives 2 × 2111/(100 × 0.42) = 101 acts per year. Assuming a mean of six casual partners per year, this is approximately 17 hook‐ups per casual partner per year.
Percent who may have concurrent partners (including group sex)	30%	Gay Community Periodic Survey 24: 30% reported currently having a regular and a casual partner concurrently. Likely to be lower bound.
Proportion of HIV‐positive MSM who inject drugs	18%	Gay Community Periodic Survey 24; Mahony et al. 9
Probability of importing an infection through injecting drug use (per week)	0.02%	Assumes that the majority of imported infections are through injecting drug use. Using an estimated 11.9% probability of infection per year for people who inject drugs 25, the probability per week found from 0.119 = 1 − (1 − prob_per_week)^52. Then assumes 18% of HIV‐positive MSM inject drugs 24.
Reduction in HCV transmission risk once HCV‐diagnosed	45%	Gay Community Periodic Survey 24: 80% disclosed STI status to any sex partner, 45% told all of their sex partners. This was implemented with the assumption that all diagnosed individuals were still able to transmit but at reduced risk (and assuming condoms were 100% effective when used).
Testing frequency HCV (times per year)	Beta (mean = 1.4, variance = 0.25)	Burnet Institute (VPCNSS): Hepatitis C Testing and Infection in HIV‐Positive Men in Melbourne, Victoria 22.
Waiting time from diagnosis to treatment commencement	Gamma (mean = 200, variance = 30)	Authors' opinion, shorter waiting time than the population average—assumes disease progression faster for co‐infected individuals 13, 44, and patients already engaged in care with some health literacy as a result of their HIV infection.
Scale‐up waiting time from diagnosis to treatment commencement	Gamma (mean = 26 weeks, variance = 5)	Assumed
Per act infection probability between serodiscordant agents	1.18% (IQR 1.10 to 1.21%)	Median and inter‐quartile range of outcomes from the calibration procedure.
Agent‐based model 2: increased heterogeneity. Where not listed parameters are the same as agent‐based model 1.		
Percent who have casual partners	90%	
Percent who may have concurrent partners (including group sex)	100%	
Number of casual partners per year	Gamma (mean = 20, variance = 46)	
Condom use probability with casual partners	Beta (mean = 10%, variance = 5%)	
Testing frequency	Beta (mean = 1.4, variance = 0.35)	Increased heterogeneity by increasing variance
Waiting time from diagnosis to treatment commencement	Gamma (mean = 200, variance = 100)	Increased heterogeneity by increasing variance
Scale‐up waiting time from diagnosis to treatment commencement	Gamma (mean = 26 weeks, variance = 10)	Increased heterogeneity by increasing variance
Per act infection probability between serodiscordant agents	0.47% (IQR 0.45 to 0.50%)	Median and inter‐quartile range of outcomes from the calibration procedure

Agent‐based model 1 is the best representation of Victorian HIV‐positive MSM population; and agent‐based model 2, which has a more heterogeneous population. IQR, inter‐quartile range; MSM, men who have sex with men; HCV, hepatitis C virus; VPCNSS, Victorian Primary Care Network on Sentinel Surveillance.

The model used weekly time steps and at each step agents in the model had an HCV status (not infected, infected and undiagnosed, diagnosed, in treatment), and could interact with other agents through “regular partnerships” (relationships lasting more than one time step), “casual partnerships” (each agent tracks a network of “fuck‐buddies”—individuals who they meet up with for casual sex on more than one occasion), or “random partnerships” (individuals who they meet up with for only a single interaction). We allowed a percentage of agents to not form any casual partnerships, and conversely a percentage of agents to have concurrent partnerships (approximating behaviours such as having one or more simultaneous partners or participating in group sex).

To account for stochastic variations in the model, multiple simulations were run. The results of 100 simulations were collated, and plots of the number of people infected with HCV, HCV incidence and number of treatments initiated was recorded over time. Median and inter‐quartile ranges (IQRs) from the simulations were compared to the outputs of the compartmental model, to determine whether more simplistic models used in data‐limited settings are likely to over‐estimate or under‐estimate outcomes.

### 2.5 ABM 2 description

**Figure 2:**
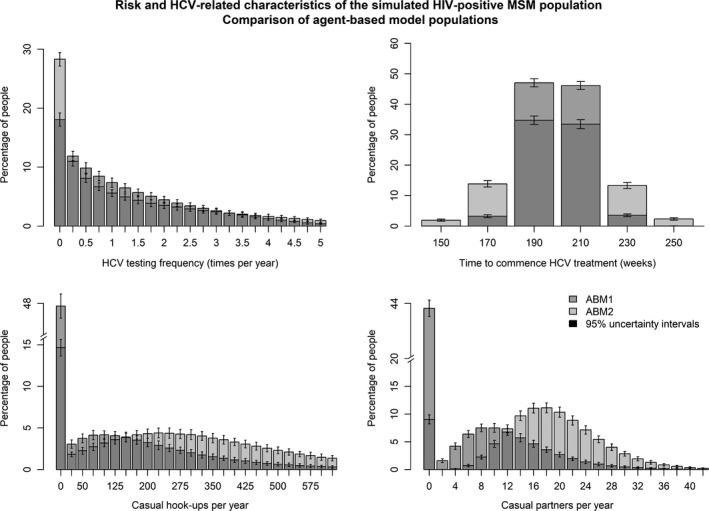
Characteristics of HIV‐positive MSM in the two agent‐based models (ABMs). ABM1 is based on local surveillance data and best estimate parameters; ABM2 is a more heterogeneous population. Distribution of testing frequency (times per year; top left), time between hepatitis C virus (HCV) diagnosis and HCV treatment commencement before treatment scale‐up (weeks; top right), the number of casual hook‐ups per year (bottom left, representing the number of times an individual hooks up with either someone in their casual partner network or a random partner) and the number of unique casual partners per year (bottom right). MSM, men who have sex with men.

A second ABM was tested, using the same approach as above but with alternate population characteristics. In this model, population heterogeneity and risk were substantially increased to represent the maximum conceivable values that a sub‐population may have. This was done to determine whether the presence of extreme heterogeneity and variations in behaviours, which may not be captured in surveillance systems, are likely to impact outcomes. In addition, the second ABM represents a situation of greater uncertainty and variation in parameters for comparison with the first (Table 2). Figure 2 compares the distributions for testing frequency, time from HCV diagnosis to treatment commencement, total number of casual sex occasions per year and number of casual partners per year between the first and second ABM populations.

### 2.6 Calibration

Each model was calibrated by varying the force of infection to achieve the desired equilibrium HCV prevalence of 10% 20 (Figure S2). For the compartmental model, this resulted in a calibrated incidence rate for the sexual transmission of HCV (i.e. excluding imported infections) among low‐ and high‐risk HIV‐positive MSM of 0.38% and 0.76% per annum respectively. For the first and second ABMs, this calibration resulted in “per‐casual‐hook‐up‐occasion” HCV transmission probabilities of 1.05% and 0.45% respectively. We were unable to find any direct estimates of this value elsewhere; however for comparison the risk of HCV transmission per injecting equipment sharing event has been estimated to be in the range 0.5% to 10% 45, 46, 47. Our calibrated estimates can also be compared to the transmission probability of HIV per receptive anal intercourse event, which has been estimated at 1.4% 48. Our values for the per‐casual‐hook‐up‐occasion sexual transmission of HCV are therefore higher than estimates for HIV. This may be due to our measure being an average over hook‐up occasions that may contain varying numbers of sexual events, and also because the HIV estimates represent an average for all MSM compared to our model population that has self‐selected as being more likely to engage in higher risk sexual practices (by virtue of having acquired HIV). Therefore, an average per‐casual‐hook‐up‐occasion HCV transmission probability among all MSM (HIV‐negative and HIV‐positive) is likely to be lower than our calibrated values.

To cross‐validate the calibrated models, the proportion of HCV‐infected people in each model who were diagnosed was compared to available data (Figure S3), and the annual HCV incidence in each model is shown against available data in Figure 5 (2015 values).

### 2.7 WHO elimination targets

The WHO elimination target aims for an 80% reduction in HCV incidence at the country level by 2030 15; however notifications among HIV‐positive MSM represent only a small proportion of new infections globally (in Victoria this is approximately 2% of all notifications 9). Since we are not modelling treatment‐as‐prevention among people who inject drugs, who represent the largest proportion of notifications 26, the impact on incidence reduction through a decline in “imported” infections is not captured by our models. For this reason a variation in the incidence reduction target was used, and we measured the time and total treatment numbers required to achieve an 80% reduction in *prevalence* among HIV‐positive MSM, in order to mimic a similar reduction in disease burden in this population. We also compared the impact of treatment scale‐up in the models against the WHO process targets of achieving 90% of people living with HCV diagnosed and 80% of people living with HCV on treatment 15.

### 2.8 Basic reproduction number (R0)

The basic reproduction number (R0)—the average number of new infections caused by one typical infected individual in a completely susceptible population—is an extremely important value for infectious diseases. Values of R0<1 indicate that the disease free state is asymptotically stable, and so the disease is expected to eventually become extinct, while values of R0>1 indicate that the disease free state is asymptotically unstable and the disease is able to invade a population 49. Therefore, we measure the R0 values for each model before treatment scale‐up and the effective reproduction number (Reff, defined as the R0 value with interventions in place) for each model after treatment scale‐up. Calculation methods for R0 and Reff are provided in the supplementary material.

### 2.9 Study dates

Modelling for this study was undertaken in 2016.

## 3 Results

### 3.1 Basic reproduction number (R0 and Reff)

Before and after treatment scale‐up the compartment model had R0 and Reff values of 1.15 and 0.42 respectively, the first ABM had R0 and Reff values of 1.32 (IQR 1.25 to 1.36) and 0.28 (IQR 0.27 to 0.29) respectively, and the second ABM had R0 and Reff values of 3.34 (IQR 3.28 to 3.41) and 0.66 (IQR 0.65 to 0.68) respectively. In all cases treatment scale‐up reduced R0 from above one to below one, indicating that with ongoing monitoring and treatment availability a rebound in new HCV infections would be unlikely.

Since all models were calibrated to the same prevalence, these findings imply there is an increasing R0 with increasing heterogeneity. This is consistent with Anderson and May 50, who found for (compartmental) models with the same R0, greater heterogeneity led to a lower prevalence and incidence throughout the course of an HIV epidemic.

### 3.2 Time to prevalence reduction

**Figure 3:**
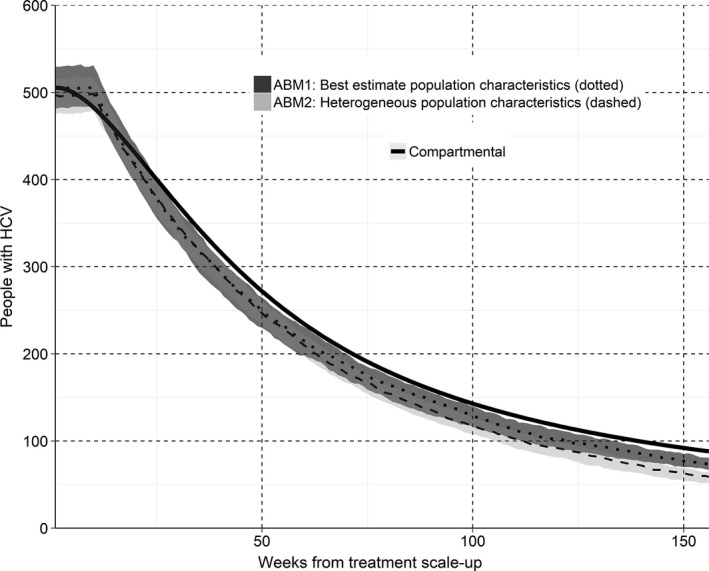
Projected number of HCV/HIV co‐infected MSM in Victoria in the first three years of treatment scale‐up. For all models the average time between HCV diagnosis and treatment commencement after scale‐up was reduced to six‐months. The solid black line represents the compartmental model; the darker (dotted) and lighter (dashed) ribbon graphs represent the median and IQR for the first ABM (parametrized by surveillance data) and the second ABM (a more heterogeneous population) respectively. IQR, inter‐quartile range; MSM, men who have sex with men; HCV, hepatitis C virus; ABM, agent‐based models.

The first ABM estimated that the prevalence of HCV among HIV‐positive MSM in Victoria could be reduced by 80% from an estimated 10% (approximately 500 HIV‐positive MSM) to below 2% (approximately 100 HIV‐positive MSM) in 122 (IQR 112 to 133) weeks if the average time from HCV diagnosis to treatment were reduced to six months (Figure 3). This was similar for the second ABM; however the compartmental model estimated that it would take approximately 139 weeks to reduce prevalence by 80%, largely due to the asymptotic decline in prevalence.

### 3.3 Treatment requirements

**Figure 4:**
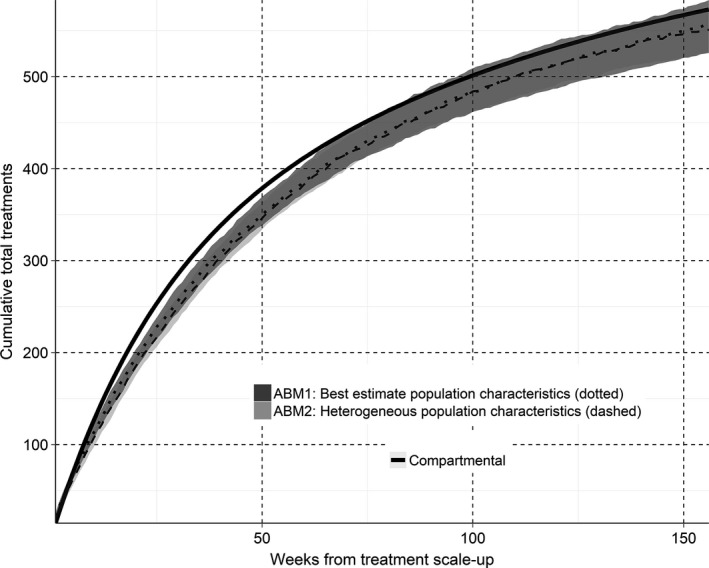
Projected cumulative number of HCV treatments provided to HCV/HIV co‐infected MSM in Victoria in over the first three years of treatment scale‐up. The solid black line represents the compartmental model; the darker (dotted) and lighter (dashed) ribbon graphs represent the median and IQR for the first ABM (parametrized by surveillance data) and the second ABM (a more heterogeneous population) respectively. IQR, inter‐quartile range; MSM, men who have sex with men; HCV, hepatitis C virus; ABM, agent‐based models.

The first ABM estimated that approximately 523 (IQR 479 to 553) treatments would be required in the first three years of treatment scale‐up to achieve an 80% HCV prevalence reduction among HIV‐positive MSM in Victoria (Figure 4). These estimates were similar to both the second ABM and the compartmental model.

### 3.4 WHO diagnosis and treatment targets

For the first ABM, it took three years following treatment scale‐up before 90% of HCV/HIV co‐infected MSM were diagnosed and four years following treatment scale‐up before 80% of HCV/HIV co‐infected MSM were treated. For the second ABM both of these targets were achieved after three years.

### 3.5 Incidence projections

**Figure 5:**
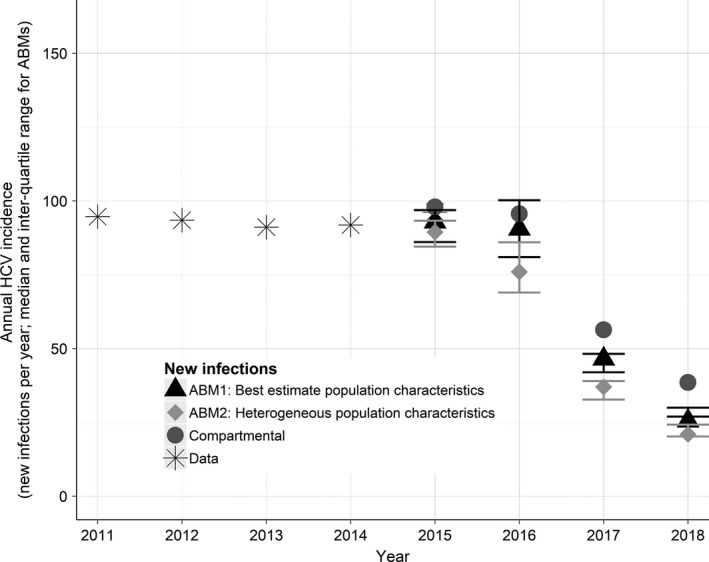
Annual incidence of HCV among HIV‐positive MSM in Victoria. Asterisks: data estimates, assuming 2% of Victorian HCV cases are among HIV‐positive MSM through sexual exposure 9, plus 18% of HIV‐positive MSM reporting injecting drug use 24 with an estimated incidence rate of 11.9% 25. Circles, triangles and diamonds: calibrated values (2015) and model projections with treatment‐scale up (2016 to 2018) for the compartmental model, first ABM (best estimates) and second ABM (heterogeneous estimates) respectively. MSM, men who have sex with men; HCV, hepatitis C virus; ABM, agent‐based models.

**Figure 6:**
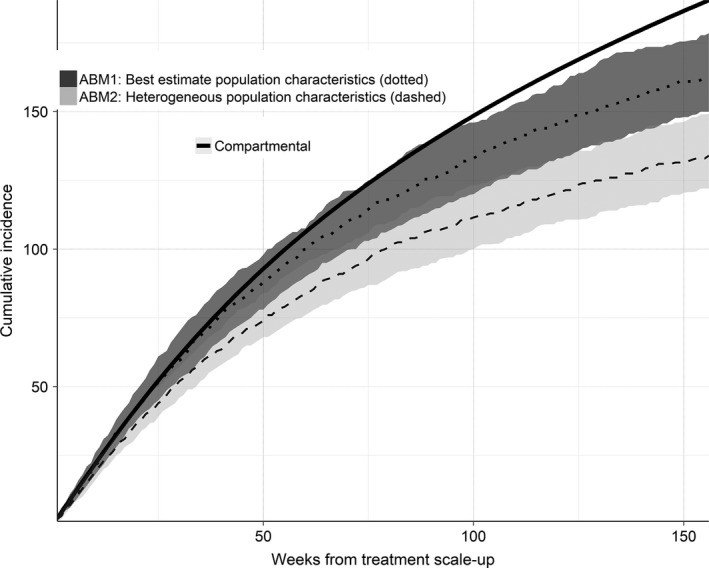
Projected cumulative incidence of HCV among HIV‐positive MSM in Victoria over the first three years of treatment scale‐up. The solid black line represents the compartmental model cumulative incidence; the darker (dotted) and lighter (dashed) ribbon graphs represent the median and IQR cumulative incidence for the first ABM (best estimates) and second ABM (more heterogeneous estimates) respectively. IQR, inter‐quartile range; MSM, men who have sex with men; HCV, hepatitis C virus; ABM, agent‐based models.

Figure 5 and Figure 6 show the projected annual and cumulative incidence of HCV respectively among HIV‐positive MSM in Victoria over the first three years of treatment scale‐up. There was a substantial difference in expected cumulative incidence between the models, with increasing heterogeneity resulting in fewer expected cumulative incident cases. As with the R0 values, this difference is consistent with Anderson and May 50: as the tail of very high‐risk individuals increases, so does the total number of risk acts in a given time period. For example, this can be seen in Figure 2 bottom‐left, which compares the total number of hook‐up occasions (within casual‐partner networks plus with random partners) between the two ABMs. In order to calibrate these models to the same observed prevalence, a lower transmission probability per hook‐up occasion was therefore required. For the bulk of the population (i.e. those not in the high‐risk tail), this meant that the average time to infection was longer, providing opportunity for a greater prevalence reduction to occur between expected transmission events, lowering the risk of acquiring HCV.

Another way of viewing this is that because higher‐risk individuals are more likely to be infected than lower‐risk individuals, as risk heterogeneity increases in the model, treatments become increasingly effective for the epidemic because they become more precisely targeted to those at greatest risk of transmission. In this sense, although the greater R0 value is detrimental to the epidemic, it is counterbalanced by an increased effectiveness of treatment‐as‐prevention through risk‐targeting. Similar arguments have been considered previously for models of other infectious diseases 51, 52, 53.

### 3.6 Sensitivity analysis

A sensitivity analysis for the compartmental model is provided in the supplementary material (Table S1).

**Figure 7:**
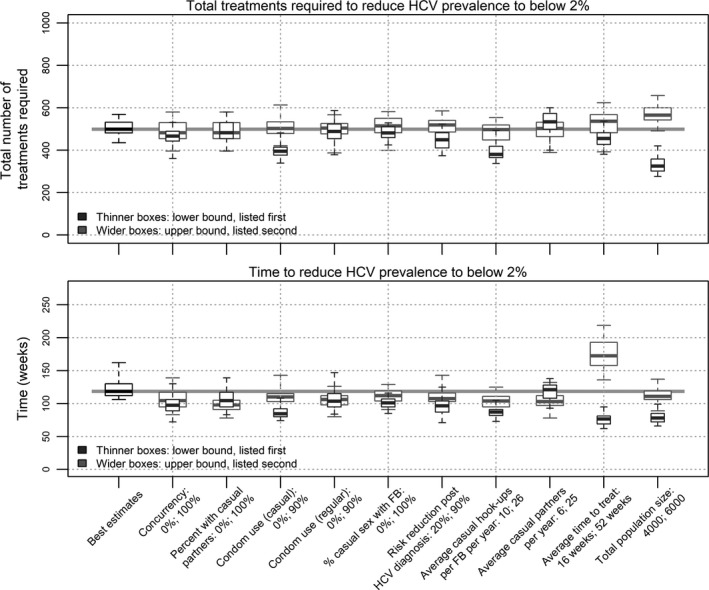
Sensitivity of the agent‐based model parameters (using ABM1). Median, inter‐quartile ranges and ranges of the total treatments and time required to reduce hepatitis C virus (HCV) prevalence among HIV‐positive men who have sex with men in Victoria to 2%. Multiple simulation runs with alternate parameter estimates for: the percentage of the model population who are able to have concurrent partners; the percentage of the model population who have one or more casual partners per year; condom use with casual partners; condom use with regular partners; the percentage of casual sex that occurs with regular‐casual partners as opposed to random‐casual partners; the reduction in risk behaviour following HCV diagnosis; the average number of hook‐ups with each regular‐casual partner per year; the average number of casual partners per year; the average time between HCV diagnosis and HCV treatment commencement following treatment scale‐up; and the total population size.

Figure 7 shows the sensitivities of parameters in the first ABM compared to base estimates for the time and number of treatments required to reduce HCV prevalence among HIV‐positive MSM to below 2%. The largest deviation in the median time and number of treatments required from multiple simulations was when condom use with casual partners, the average time from diagnosis to commencing treatment and the total population size were varied. The influence of time from diagnosis to treatment commencement highlights the importance of patient follow‐up and engagement in HCV care. In particular, if the average time from HCV diagnosis to treatment commencement could be reduced to 16 weeks, reducing prevalence by 80% was possible in a median of 77 (IQR 69 to 81) weeks with only 455 (IQR 427 to 482) treatments. Otherwise, outcomes were fairly robust to changes in behavioural parameters, which may explain why the confidence intervals for the two ABMs in Figures 3, 4, 5, 6 to were similar in size, despite the second ABM having more highly skewed behavioural parameter distributions.

A Latin Hypercube uncertainty analysis was conducted to determine the extent of multivariate uncertainty, and is provided in the supplementary material (Figure S5).

## 4 Discussion

We used multiple modelling techniques to estimate the time and treatment numbers required to achieve an 80% reduction in HCV prevalence among HIV‐positive MSM in Victoria. Using an ABM parametrized with local surveillance data and best available estimates of sexual risk behaviour, this prevalence reduction could be achieved in approximately 122 (IQR 112 to 133) weeks with approximately 523 (IQR 479 to 553) treatment episodes if the average time from HCV diagnosis to treatment was reduced from approximately four years (pre‐DAAs) to six months (post‐DAAs). This is slightly more than the total number of HCV/HIV co‐infected MSM due to the occurrence of new and re‐infections during this period. Furthermore, we found that this prevalence reduction could be achieved in only 77 (IQR 69 to 81) weeks if the average time between diagnosis and treatment commencement was reduced to only 16 weeks.

The influence of time from diagnosis to treatment commencement on HCV transmission in the model highlights the importance of routine HCV monitoring and education as a part of HIV care, and rapid treatment commencement for those newly diagnosed with HCV. Given the high tolerability of DAA treatments for HCV and the Australian government's commitment of AUS$1 billion over five years for unlimited and unrestricted DAA treatment access 54, 55, 56, rapid commencement of treatment should be possible. Our model suggests that this could have substantial effects on the HCV epidemic among HIV‐positive MSM. Furthermore, although these models have considered co‐infection with HCV, the importance of monitoring and rapid treatment commencement is generalizable to other sexually transmitted infections that have a similar prevalence among this population, for example syphilis and gonorrhoea.

Compartmental models are typically used to approximate HCV transmission because data to parametrize network‐based or ABMs is often unavailable for the desired setting. Our multi‐modelling methodology found consistent outcomes between the compartmental and ABM approaches for the time and treatment requirements, which supports the use of compartments models for resource allocation decisions. However, when considering novel approaches to disease control that may target specific behaviours or individuals, for example network‐based treatment approaches 57 or contact tracing 58, more sophisticated models will be required, as compartmental models that approximate these effects have been shown to break down in these circumstances 59, 60, 61, 62.

When an alternate ABM was used, which incorporated greater uncertainty and more highly skewed transmission risk behaviours, estimates for the time and treatment numbers needed to achieve an 80% reduction in prevalence were not significantly different from the first ABM; however there were fewer HCV transmission events. This suggests that for two populations with the same prevalence, HCV treatment‐as‐prevention may be more effective in the population with a greater degree of risk heterogeneity. Given that sexual risk behaviour among HIV‐positive MSM is known to be highly heterogeneous 43 and data sources may underestimate this heterogeneity due to limited data capture or reporting bias, estimates of HCV treatment‐as‐prevention in this group are likely to be conservative. This is particularly true of compartmental models which are generally limited to a small number of discrete risk levels.

Using an ABM to link distributions of behaviour with disease transmission dynamics we have identified which behaviours are most likely to influence HCV transmission and elimination among HIV‐positive MSM. In our model, the frequency and consistency of condom use with casual partners was associated with the greatest uncertainties in epidemic predictions. Therefore, obtaining reliable estimates for these parameters among HIV‐positive MSM should be prioritized in future studies, in particular due to the current paucity of data.

The models used in this study have a number of limitations, largely due to limitations in their parameter sources and lack of epidemiological data to inform additional features. First, the model parameters were derived from a variety of sources that may not accurately represent all HIV‐positive MSM in Victoria. For this reason we tested alternate modelling methods and scenarios, in particular of a highly heterogeneous population, and conducted sensitivity analyses to study the influence of parameters on HCV transmission and prevalence reduction. Our projections were consistent using multiple modelling approaches, providing confidence in our estimates. Second, this work has been designed to test the impact of different model assumptions about sexual risk behaviours on the sexual transmission of HCV. Although we have been able to assess a number of assumptions, there are many complexities of sexual behaviour that were not included in the ABMs due to a lack of epidemiological data; for example changes in casual (“fuck‐buddy”) networks among MSM over time, HCV sero‐sorting, age‐assortative mixing and different types of sexual activity that we were subsequently unable to investigate. Similarly, due to a lack of injecting network epidemiological data among this population we could not assess the interaction of sexual networks with injecting networks. Therefore, we approximated incidence through injecting networks as a constant flow of “imported infections”; however in the case of a broader country‐level HCV elimination strategy, treatment‐as‐prevention among people who inject drugs may reduce non‐sexual HCV transmission to HIV‐positive MSM over time, which would decrease the time and treatment numbers required to reach the prevalence reduction target. Informing these gaps in data is an important area for future work, as our analysis has shown that different assumptions about transmission networks can have important implications. Third, these models do not include HIV and HCV disease progression or mortality. Given the time frame being projected was only three years, changes in disease stage during this period are unlikely to significantly impact sexual transmission. Moreover, in Australia 83% of HIV‐positive MSM are estimated to be enrolled in anti‐retroviral therapy (ART). This high level of engagement in care means that the universal access to DAA treatments for HCV from 2016 is expected to reduce HCV‐related mortality among this population to be negligible (and possibly zero) over the time frame considered. It is also possible that MSM who are on ART have a lower probability of sexually acquiring HCV; however as parametrization data were not available by ART status, these effects were averaged as part of the calibration procedure to obtain population‐level forces of infection. Given that dramatic changes to ART coverage in Australia are unlikely over the projection period this is not expected to influence outcomes.

## 5 Conclusions

Major reductions in HCV prevalence can be achieved among HCV/HIV co‐infected MSM within two years. Uncertainties surrounding the distribution of risk behaviours within this population are likely to make resource requirement estimates conservative, as treatment‐as‐prevention may be more effective in highly heterogeneous MSM populations. In particular, compartmental modelling approaches are likely to provide reasonable approximations for settings where detailed behavioural data are unavailable. Future epidemiological studies should focus on measuring the frequency and consistency of condom use with casual partners, as this was the behavioural factor most likely to influence epidemic projections among MSM.

## Competing interests

JD, MH and the Burnet Institute receive investigator‐initiated research funding from Gilead Sciences, AbbVie and BMS. JD's institution has received honoraria from Merck, Gilead and BMS. No pharmaceutical grants were received in the development of this study.

## Authors' contributions

NS performed the modelling and drafted the manuscript. NS, JD and MH conceived the study. MS and CEH provided behavioural parameter estimates. DW and OK critically reviewed the modelling. All authors contributed to model interpretation. All authors were involved in revising the manuscript.

## Supporting information


**Figure S1.** Compartmental model schematic.

**Figure S2.** Prevalence of HCV among HIV‐positive MSM in Victoria. Comparison of data estimate to outcomes of the calibrated compartmental model, the first ABM (best estimates) and the second ABM (more heterogeneous estimates). Values for the ABMs represent medians and inter‐quartile ranges of all simulations.

**Figure S3.** Proportion of people living with HCV who are diagnosed. Comparison of data estimate to outcomes of the calibrated compartmental model, the first ABM (best estimates) and the second ABM (more heterogeneous estimates). Values for the ABMs represent medians and inter‐quartile ranges of all simulations.

**Figure S4.** Projected incidence of HCV among HIV‐positive MSM in Victoria over the first three years of treatment scale‐up. The blue and red scatter plots represent median and inter‐quartile ranges (IQRs) of the weekly incidence after multiple simulations for the first ABM (best estimates) and second ABM (more heterogeneous estimates) respectively.

**Figure S5.** Latin Hypercube uncertainty analysis. Blue boxplots: Variation in the average (after 10 simulations) time and treatment numbers required to reduce HCV prevalence among HIV‐positive MSM by 80%, as parameters move through points on the Latin Hypercube. Performed for risk population‐related parameters (proportion who have casual partners, proportion who have concurrent partners), frequency‐related parameters (average number of casual partners per year, average number of hook‐ups per fuck buddy per year, percent of casual sex with partners outside of fuck buddy network), and risk reduction‐related parameters (condom use among casual partners, condom use among regular partners, risk reduction following HCV diagnosis). Black boxplot: stochastic variation from 100 simulations with point estimate parameters.

**Table S1.** Sensitivity analysis for the compartmental model. Impact of alternate parameters on estimates of the time and treatment numbers required to reduce HCV prevalence among HIV‐positive MSM by 80%
