# Supplementary figures and images for: Eliminating hepatitis C virus as a public health threat among HIV‐positive men who have sex with men: a multi‐modelling approach to understand differences in sexual risk behaviour
Source: J Int AIDS Soc. 2018 Jan 9;21(1):e25059. doi: 10.1002/jia2.25059 (PMC5810343; doi:10.1002/jia2.25059)

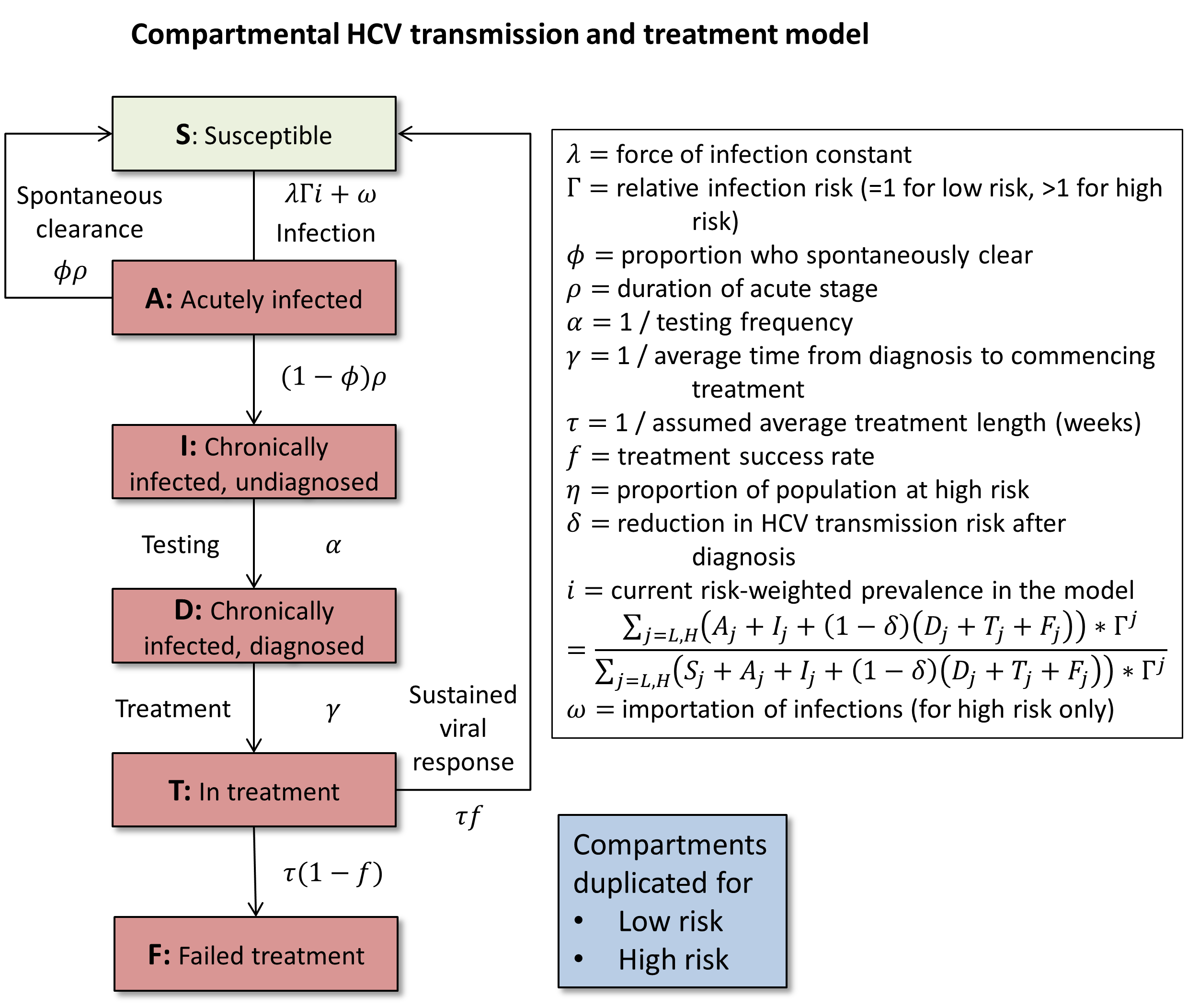

Supplement: Supplementary file 1 — Figure S1. Compartmental model schematic. [file JIA2-21-e25059-s001.tif]

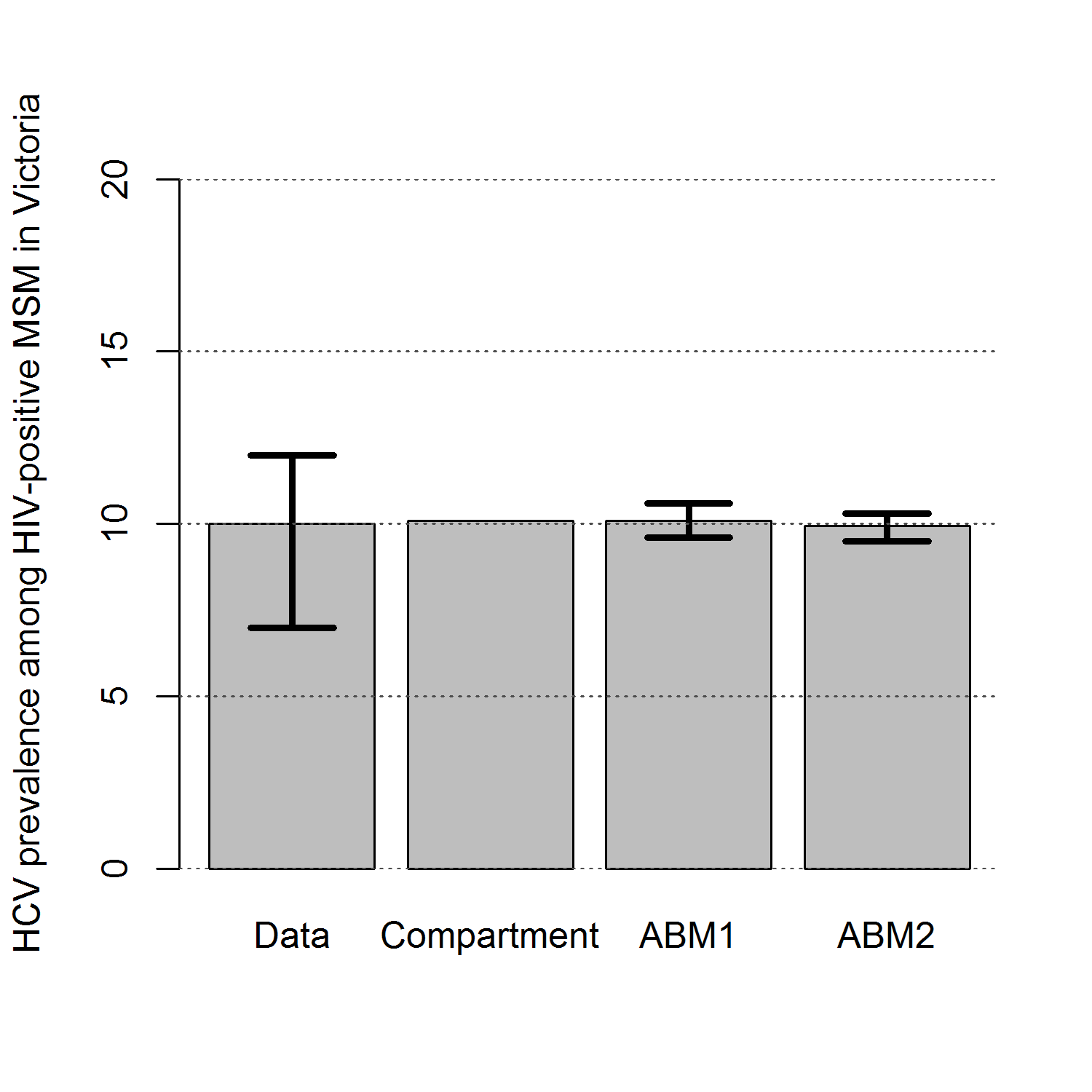

Supplement: Supplementary file 2 — Figure S2. Prevalence of HCV among HIV‐positive MSM in Victoria. Comparison of data estimate to outcomes of the calibrated compartmental model, the first ABM (best estimates) and the second ABM (more heterogeneous estimates). Values for the ABMs represent medians and inter‐quartile ranges of all simulations. [file JIA2-21-e25059-s002.tiff]

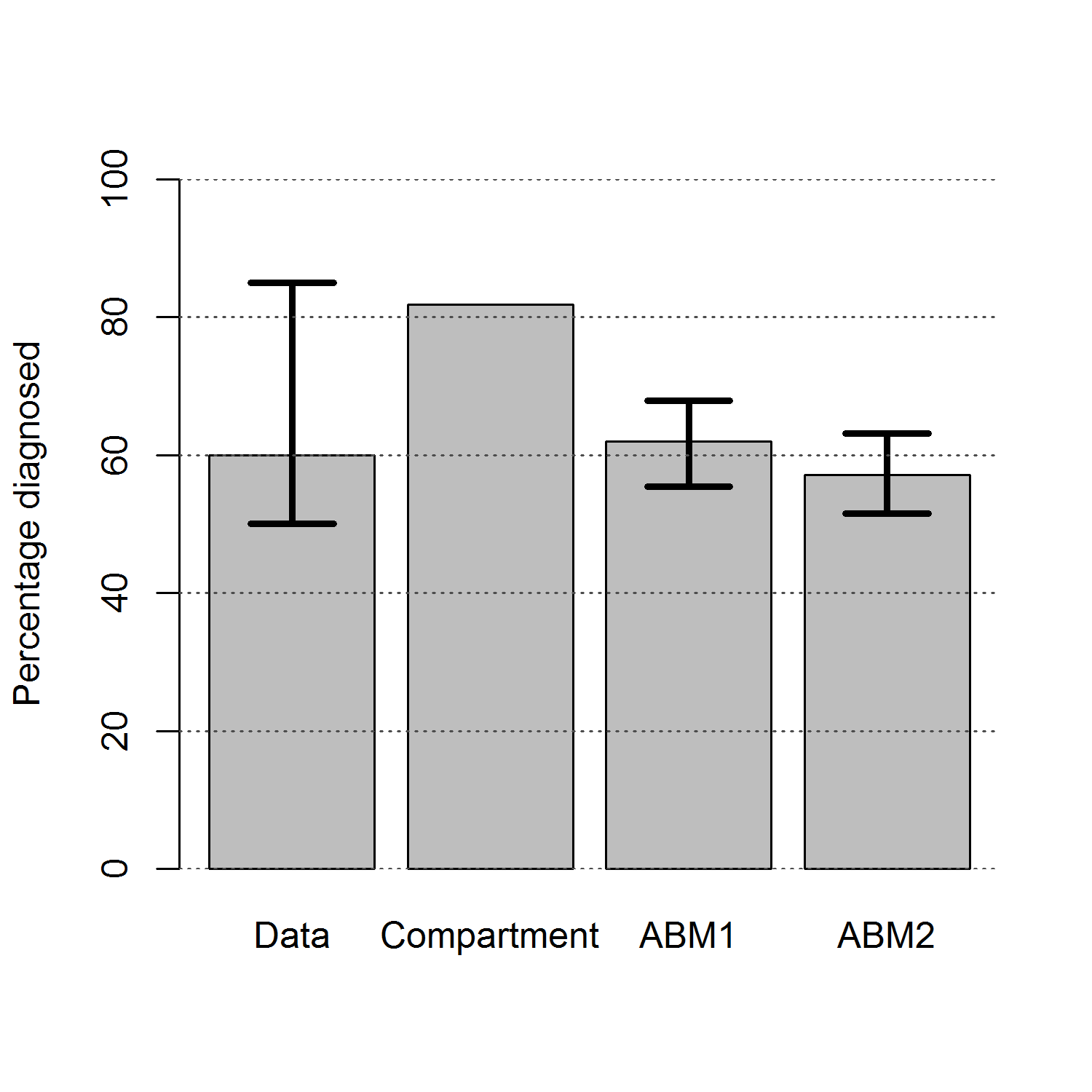

Supplement: Supplementary file 3 — Figure S3. Proportion of people living with HCV who are diagnosed. Comparison of data estimate to outcomes of the calibrated compartmental model, the first ABM (best estimates) and the second ABM (more heterogeneous estimates). Values for the ABMs represent medians and inter‐quartile ranges of all simulations. [file JIA2-21-e25059-s003.tiff]

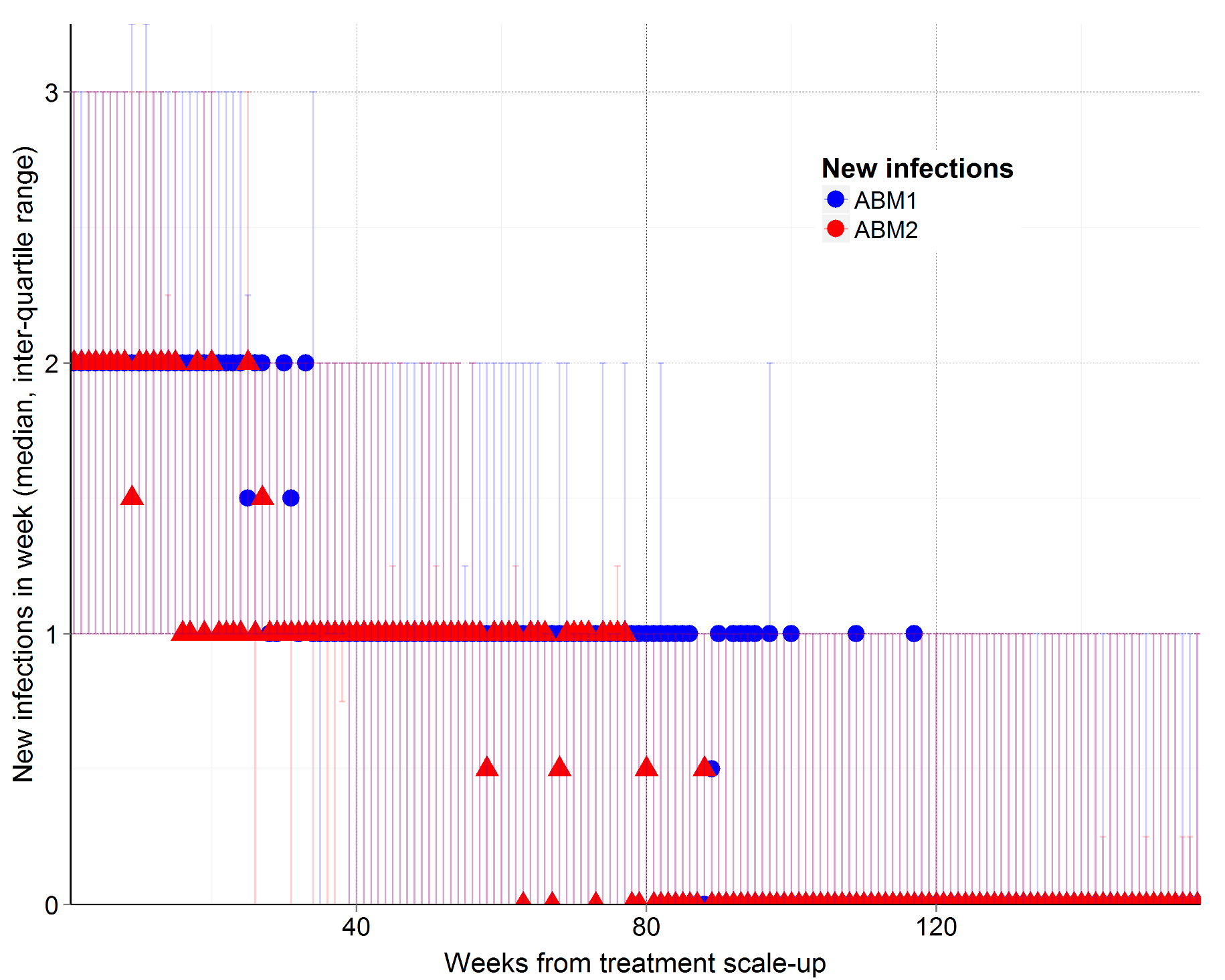

Supplement: Supplementary file 4 — Figure S4. Projected incidence of HCV among HIV‐positive MSM in Victoria over the first three years of treatment scale‐up. The blue and red scatter plots represent median and inter‐quartile ranges (IQRs) of the weekly incidence after multiple simulations for the first ABM (best estimates) and second ABM (more heterogeneous estimates) respectively. [file JIA2-21-e25059-s004.tiff]

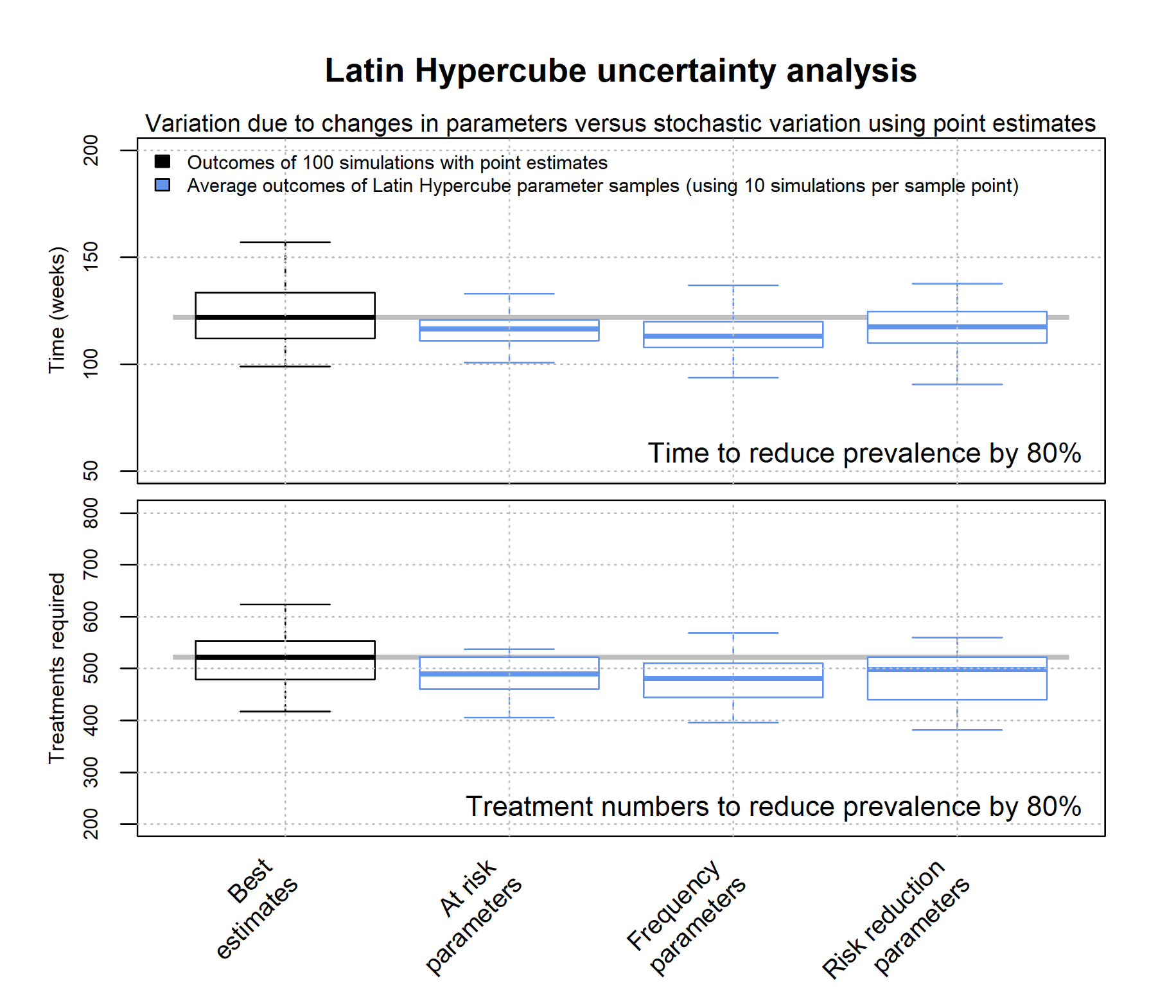

Supplement: Supplementary file 5 — Figure S5. Latin Hypercube uncertainty analysis. Blue boxplots: Variation in the average (after 10 simulations) time and treatment numbers required to reduce HCV prevalence among HIV‐positive MSM by 80%, as parameters move through points on the Latin Hypercube. Performed for risk population‐related parameters (proportion who have casual partners, proportion who have concurrent partners), frequency‐related parameters (average number of casual partners per year, average number of hook‐ups per fuck buddy per year, percent of casual sex with partners outside of fuck buddy network), and risk reduction‐related parameters (condom use among casual partners, condom use among regular partners, risk reduction following HCV diagnosis). Black boxplot: stochastic variation from 100 simulations with point estimate parameters. [file JIA2-21-e25059-s005.tiff]
