# Supplementary material for: Eliminating hepatitis C virus as a public health threat among HIV‐positive men who have sex with men: a multi‐modelling approach to understand differences in sexual risk behaviour
Source: J Int AIDS Soc. 2018 Jan 9;21(1):e25059. doi: 10.1002/jia2.25059 (PMC5810343; doi:10.1002/jia2.25059)
Supplement: Supplementary file 6 — Table S1. Sensitivity analysis for the compartmental model. Impact of alternate parameters on estimates of the time and treatment numbers required to reduce HCV prevalence among HIV‐positive MSM by 80% [file JIA2-21-e25059-s006.pdf]

Supplementary material

Eliminating hepatitis C virus as a public health threat among HIV-positive men who have sex with men: a multi-modelling approach to understand differences in sexual risk behaviour

Detailed compartmental model description

We used a dynamic compartmental model to approximate HCV transmission, diagnosis and treatment among HIV-positive MSM as shown in Figure S1, with parameters and their sources provided in Table 1. The population was categorised as either: susceptible (i.e. not HCV-infected) (S), Acutely HCV-infected (A), chronically HCV-infected and not diagnosed (I), chronically HCV-infected and diagnosed (D), in treatment for HCV (T), or failed treatment for HCV (F). Entry into and exit from the population was not included given the short three-year time frame being modelled to achieve elimination. Each compartment was stratified by high and low levels of risk, with high-risk individuals having their risk of acquiring or transmitting HCV increased by a factor of Γ compared to low-risk individuals. Individuals did not cycle between risk categories as risk states have been found to change little in the time frame being considered [1]. Once diagnosed, individuals reduced their risk of onward transmission by a factor δ.

When the model was run susceptible MSM became infected at a rate $\beta = \lambda i$ if they were at low risk or $\beta = \lambda \Gamma i + \omega$ if they were at high risk, where λ is a constant, i is the current risk-weighted HCV prevalence in the model and ω is the infection import parameter. If the subscripts L and H represent low risk and high risk respectively, and $\Gamma^L = 1$, then

$$i = \frac{\sum_{j=L,H}\left(I_j + (1-\delta)(D_j + T_j + F_j)\right) * \Gamma^j}{\sum_{j=L,H}\left(S_j + I_j + (1-\delta)(D_j + T_j + F_j)\right) * \Gamma^j}.$$

Spontaneous clearance of HCV-infection occurred for a proportion ϕ of people following an average duration $1/\rho$ weeks in the acute stage of infection, while the remaining $(1-\phi)$ became chronically infected and moved to the I compartment. After an average duration $1/\alpha_L$ weeks in the I compartment ($1/\alpha_H$ weeks for the high risk population), people were tested and diagnosed, progressing to the D compartment, where they spent an average $1/\gamma_L$ weeks ($1/\gamma_H$ weeks for high risk) before commencing treatment and moving to the T compartment. Treatment was assumed to last an average of $1/\tau$ weeks, before a proportion f achieved a sustained viral response and moved back to the S compartment. The proportion $(1-f)$ who failed treatment were moved to the F compartment where they remained. Parameters and their sources are provided in Table 1.

Calibration

The model started with a population size of 5000 (the estimated number of HIV positive MSM in Victoria [2]) and the infection parameter λ was varied until the equilibrium prevalence was 10% [3]. As a closed population was modelled, to avoid an unrealistic build-up in the F compartment during the model burn-in period the proportion f achieving a sustained viral response was assumed to be 1 for calibration (i.e. before equilibrium was reached and the simulations started).

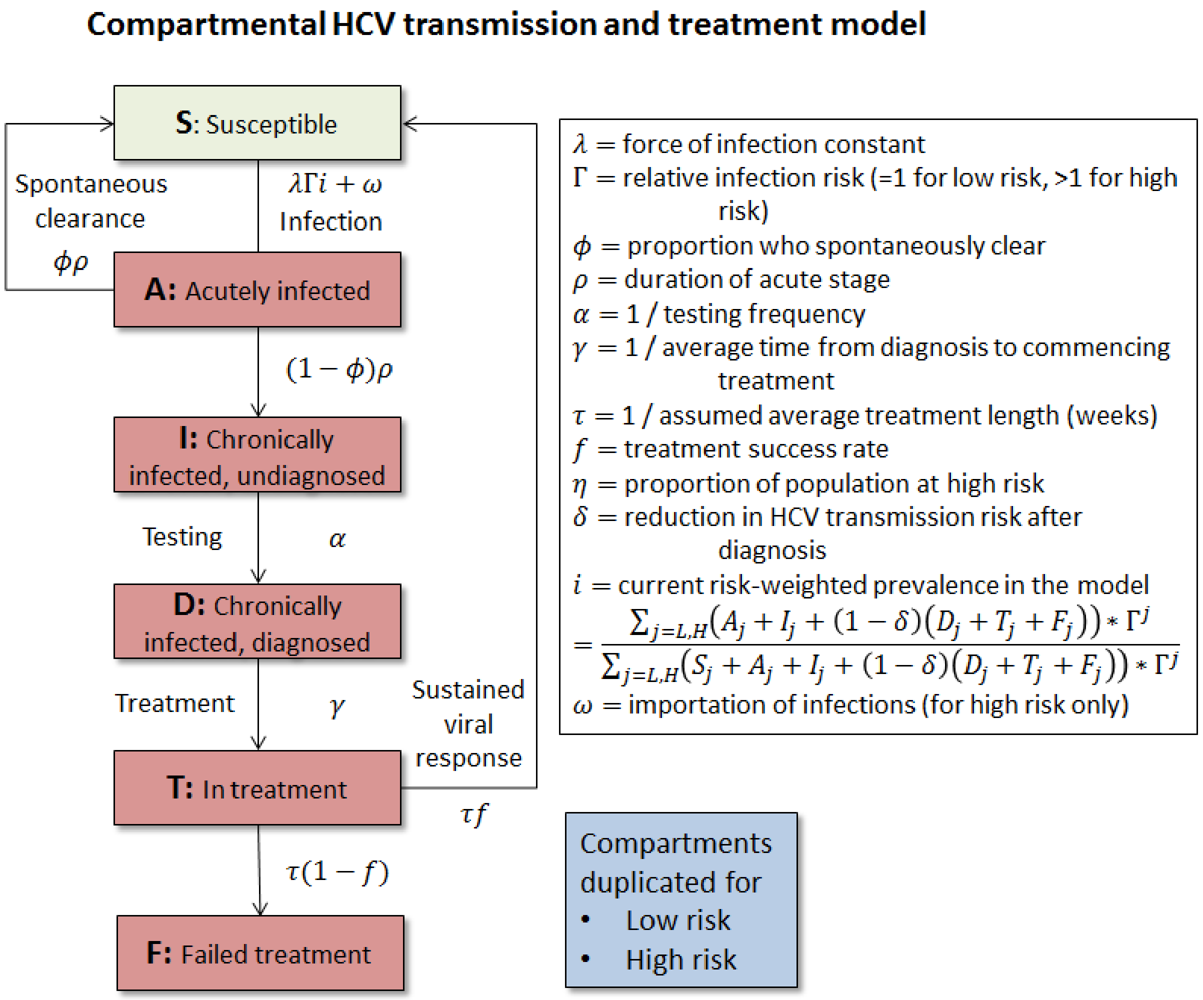

Figure S1: Compartmental model schematic

Equations

The model is described by the following set of differential equations, where the L and H subscripts represent the low and high risk populations respectively.

Low risk population:

$$\frac{dS_L}{dt} = -\lambda i S_L + \rho\phi A_L + \tau f T_L$$

$$\frac{dA_L}{dt} = \lambda i S_L - \rho A_L$$

$$\frac{dI_L}{dt} = \rho(1-\phi)A_L - \alpha I_L$$

$$\frac{dD_L}{dt} = \alpha I_L - \gamma D_L$$

$$\frac{dT_L}{dt} = \gamma D_L - \tau T_L$$

$$\frac{dF_L}{dt} = \tau(1-f)T_L$$

High risk population:

$$\frac{dS_H}{dt} = -(\lambda \Gamma \mathrm{i} + \omega)S_H + \rho\phi A_H + \tau f T_H$$

$$\frac{dA_H}{dt} = (\lambda \Gamma i + \omega)S_H - \rho A_H$$

$$\frac{dI_H}{dt} = \rho(1-\phi)A_H - \alpha I_H$$

$$\frac{dD_H}{dt} = \alpha I_H - \gamma D_H$$

$$\frac{dT_H}{dt} = \gamma D_H - \tau T_H$$

$$\frac{dF_H}{dt} = \tau(1-f)T_H$$

Where the dynamic risk-weighted prevalence, i, is given by

$$i = \frac{\sum_{j=L,H}\left(A_j + I_j + (1-\delta)\left(D_j + T_j + F_j\right)\right) * \Gamma^j}{\sum_{j=L,H}\left(S_j + A_j + I_j + (1-\delta)(D_j + T_j + F_j)\right) * \Gamma^j}.$$

Additional agent-based model details

In addition to the variables in Table 2, agents had the following counter / tracker variables:

- HCV-infection status (0 or 1)
- Length of HCV infection (0 if not infected or number of weeks if infected)
- Diagnosis status (0 if not infected or 1 if infected)
- Regular-partnership status (0 or 1)
- Regular-partner (agent in model who is their current regular-partner)
- Regular-partnership length (0 or number of weeks in current regular-partnership)
- Regular-casual partner network (a list of agents who are repeated casual partnerships)
- Time on treatment (0 if not on treatment or 1-17 if being treated)

Simulation steps

The model time-steps represent one week, and at each step the following eight procedures are performed:

1. ***Increase the duration of existing infections, partnerships and treatment courses:***
   Agents who reach 17 weeks on treatment (see Table S1) become uninfected (setting HCV-infection status=0, length of infection=0, diagnosis status=0 and time on treatment=0).
2. ***Create new regular-partnerships:***
   For each agent with no regular-partner, an independent random number pr< 52 is drawn. If pr<(regular partners per year) AND there is another un-partnered agent with pr<(partners per year), the two agents will pair (both setting partnership status=1 and each other as their partner).
3. ***End some old regular-partnerships:***
   For each regular-partnership, if the partnership length > the average relationship length of either agent then a random number pr<1 is drawn. If pr< (1 / difference in average relationship lengths of partners), then they will separate (both setting partnership status=0 and regular partner to nobody).
4. ***Assign some casual interactions:***
   For each agent who is available for casual sex (i.e. either does not have a regular partner or has a regular partner and is able to have concurrent partners), an independent random number pr<52 is drawn. If pr<frequency of hook-up with a casual partner, the agent will create a temporary casual-partnership link to either an available agent in their regular-casual network (if another random number pr2 < casual partners per year*(1- % of casual hook-ups that are with regular partners)) or to a randomly selected available agent.
5. ***Spread infection through some discordant partnerships:***
   For each partnership link (regular or casual), an independent random number pr<100 is drawn. If pr>the condom use probability of either agent, condoms are assumed to have been used inconsistently. Therefore, to determine the chance of infection spread a second random number pr2<100 is drawn. If pr2<infection-chance (the global parameter, see calibration below) then the uninfected partner acquires HCV (setting HCV infection status=1).
6. ***End casual interactions:***
   Links assigned in step 5 will be removed. This does not change the regular-casual network of individual agents.
7. ***Allow agents to test for HCV-infection:***
   For each HCV-infected agent, an independent random number pr<52 is drawn. If pr<their test frequency then the agent is diagnosed (setting diagnosis status=1).

8. ***Allow diagnosed HCV-infected agents to commence treatment:***
   For each infected and diagnosed agent, an independent random number pr<52 is drawn. If pr< their waiting time from diagnosis to treatment commencement then the agent commences treatment (setting time on treatment=1).

Calibration

A similar approach to calibration was taken as for the compartmental model; namely, the "infection chance parameter" – the probability that HCV is transmitted between a discordant MSM partnership in a single time step – was varied until the estimated HCV prevalence of 10% among HIV-positive MSM in Victoria was achieved. However, due to the stochasticity of the model this involved incremental variations of the "infection chance" parameter throughout an extensive model burn-in period. The model was started with 10% of the population infected and undiagnosed. Every 30 time-steps, the prevalence was checked: if it was greater than 11.5%, the infection chance probability was lowered by 10% of its current value, if it was less than 8.5%, the infection chance probability was increased by 10% of its current value, and if it fell within the accepted range of 8.5–11.5%, no changes were made. The model was then run for another 30 time steps and this process was repeated. This was continued until the model prevalence had been within the 8.5–11.5% range for 15 consecutive checks, as which point the simulation burn-in period was ended.

Implementing treatment scale-up

After the burn-in period of a simulation was completed, the testing rates and waiting time to treatment variables were re-distributed with values drawn (independently for each agent) from the intervention distributions shown in Table S2. The number of people infected with HCV and cumulative number of treatments initiated was recorded at each time step for a three-year projection.

The basic reproduction number (R0)

Compartmental model

We use the methods from Diekmann et al. [4]—see also [5]—to calculate the basic reproduction number (R0) (and similarly the effective reproduction number [Reff]) of our system before and after

treatment scale-up. This was calculated as the spectral radius (maximum of the absolute value of eigenvalues) of the next generation matrix. Let:

- $x = (A_L, A_H, I_L, I_H, D_L, D_H, T_L, T_H, S_L, S_H)^T$ be the vector of compartments;
- $\mathcal{F}_k(x)$ be the rate of appearance of new infections in compartment k (i.e. individuals entering the kth element of x *only as a result of infection*);
- $\mathcal{V}_k$ be the net rate individuals transfer out of compartment k by all means aside from new infection (i.e. $\mathcal{V}_k$ is the number of individuals exiting compartment k minus the number of individuals entering compartment k, through means other than infection); and
- x_0 be the size of each compartment in the disease free equilibrium state.

The only compartments with non-zero numbers of new infections entering are A_L and A_H so that the relevant values are:

- $\mathcal{F}_1 = \lambda i S_L$
- $\mathcal{F}_2 = (\lambda \Gamma \mathrm{i} + \omega) S_H$
- $\mathcal{V}_1 = \rho A_L$
- $\mathcal{V}_2 = \rho A_H$
- $\mathcal{V}_3 = \alpha I_L - (1 - \phi)\rho A_L$
- $\mathcal{V}_4 = \alpha I_H - (1 - \phi)\rho A_H$
- $\mathcal{V}_5 = -\alpha I_L + \gamma D_L$
- $\mathcal{V}_6 = -\alpha I_H + \gamma D_H$
- $\mathcal{V}_7 = -\gamma D_L + \tau T_L$
- $\mathcal{V}_8 = -\gamma D_H + \tau T_H$
- $x_0 = (0,0,0,0,0,0,0,0, N_L, N_H,)^T$
- $N_L = 5000 * (1 - \eta), N_H = 5000 * \eta$

Then from [4, 5], R0 will be the spectral radius of

$$\left[\frac{\partial \mathcal{F}_k}{\partial x_j}(x_0)\right]\left[\frac{\partial \mathcal{V}_k}{\partial x_j}(x_0)\right]^{-1}$$

$$= \frac{\lambda}{(N_L + \Gamma N_H)} \begin{bmatrix} N_L & \Gamma N_L & N_L & \Gamma N_L & \delta N_L & \Gamma\delta N_L & \delta N_L & \Gamma\delta N_L \\ \Gamma N_H & \Gamma^2 N_H & \Gamma N_H & \Gamma^2 N_H & \Gamma\delta N_H & \Gamma^2\delta N_H & \Gamma\delta N_H & \Gamma^2\delta N_H \\ 0 & 0 & 0 & 0 & 0 & 0 & 0 & 0 \\ 0 & 0 & 0 & 0 & 0 & 0 & 0 & 0 \\ 0 & 0 & 0 & 0 & 0 & 0 & 0 & 0 \\ 0 & 0 & 0 & 0 & 0 & 0 & 0 & 0 \\ 0 & 0 & 0 & 0 & 0 & 0 & 0 & 0 \\ 0 & 0 & 0 & 0 & 0 & 0 & 0 & 0 \end{bmatrix}$$

$$* \begin{bmatrix} \rho & 0 & 0 & 0 & 0 & 0 & 0 & 0 \\ 0 & \rho & 0 & 0 & 0 & 0 & 0 & 0 \\ -(1-\phi)\rho & 0 & \alpha & 0 & 0 & 0 & 0 & 0 \\ 0 & -(1-\phi)\rho & 0 & \alpha & 0 & 0 & 0 & 0 \\ 0 & 0 & -\alpha & 0 & \gamma & 0 & 0 & 0 \\ 0 & 0 & 0 & -\alpha & 0 & \gamma & 0 & 0 \\ 0 & 0 & 0 & 0 & -\gamma & 0 & \tau & 0 \\ 0 & 0 & 0 & 0 & 0 & -\gamma & 0 & \tau \end{bmatrix}^{-1}$$

which has eigenvalues 0 and $\frac{\lambda}{N_L + \Gamma N_H}(N_L + \Gamma^2 N_H)\left(\frac{1}{\rho} + (1-\phi)\left(\frac{1}{\alpha} + \frac{1-\delta}{\gamma} + \frac{1-\delta}{\tau}\right)\right)$.

Therefore

$$R0 = \frac{\lambda}{N_L + \Gamma N_H}(N_L + \Gamma^2 N_H)\left(\frac{1}{\rho} + (1-\phi)\left(\frac{1}{\alpha} + \frac{1-\delta}{\gamma} + \frac{1-\delta}{\tau}\right)\right)$$

Agent-based model

A similar approach was used for the agent-based model (ABM), following Anderson and May [6]. Let:

- "Regular partners or not" be a binary variable for whether or not an individual is in a regular relationship;
- "Concurrency" be a binary variable for whether or not an individual has concurrent partners (including when in a regular relationship); and
- "m" be the expected proportion of a year that an individual spends in a monogamous regular relationship.

Then

$$\mathrm{m} = \min(1, \text{regular parners or not} * \text{average regular relationship length}) * (1 - \text{concurrency})$$

and we can calculate an individual's expected number of transmission (if infected) as:

$$\begin{aligned} \text{c} = \ & \text{force of infection} * \text{expected duration of infection} \\ & * [\text{m} * (1 - \text{condom use regular}) * \text{average regular relationship length} + (1 - \text{m}) \\ & * (1 - \text{condom use casual}) * \text{ number of casual partners per year} \\ & * \text{average hook– ups per year with each casual partner}] \end{aligned}$$

where the expected duration of infection for an individual is weighted by their transmission risk and defined as

$$\begin{aligned} & \text{proportion who spontaneously clear} * \text{duration of acute stage} \\ & + \text{proportion who do not spontaneously clear} \\ & * \left(\text{duration of acute stage} + \frac{1}{\text{testing frequency}} \right. \\ & \left. + (1 - \text{risk reduction post diagnosis}) * (\text{time from diagnosis to treatment}) \right). \end{aligned}$$

If we let Pr(c) be the probability density function for c, then the expected number of new infections caused by one typical infected individual in a completely susceptible population is calculated as

$$R0 = \int c\mathrm{Pr}(c) \approx \frac{1}{N}\sum_{j=1}^{N} c$$

for a population size N.

Additional figures

Figure S2 compares the calibrated prevalence for each of the models (median and interquartile range [IQR] for the ABMs) against the desired equilibrium HCV prevalence of 10% [3]

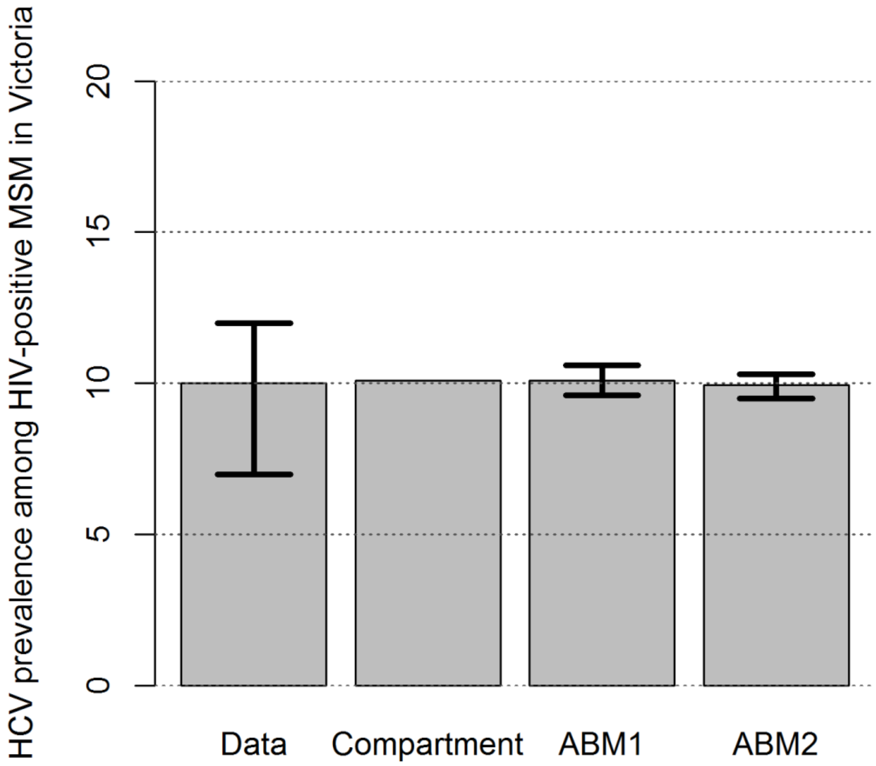

Figure S2: Prevalence of HCV among HIV-positive MSM in Victoria. Comparison of data estimate to outcomes of the calibrated compartmental model, the first ABM (best estimates) and the second ABM (more heterogeneous estimates). Values for the ABMs represent medians and inter-quartile ranges of all simulations.

Figure S3 compares the calibrated model values for the percentage of people living with HCV who have been diagnosed against data estimates. Data estimates were taken from surveillance reports estimating that 85% of people living with HCV in Australia have had an antibody test [7]. However, HCV antibodies could be present due to acute, chronic or resolved infection, and so a follow-up RNA tests is required to constitute a complete diagnosis. It has been estimated that 50–70% of people living with HCV have had both an antibody and RNA test [8].

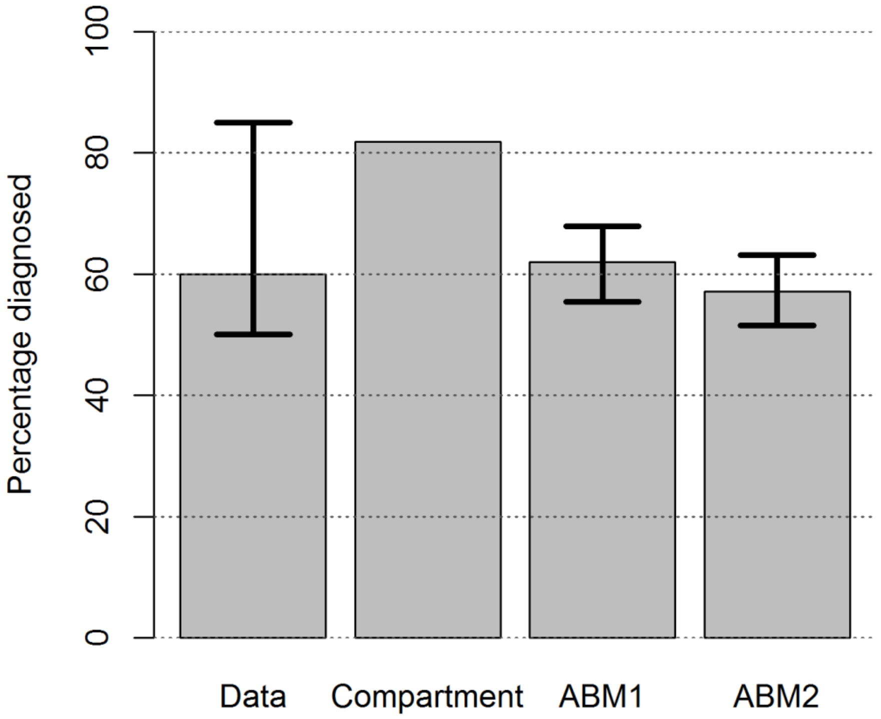

Figure S3: Proportion of people living with HCV who are diagnosed. Comparison of data estimate to outcomes of the calibrated compartmental model, the first ABM (best estimates) and the second ABM (more heterogeneous estimates). Values for the ABMs represent medians and inter-quartile ranges of all simulations.

Figure S4 shows the weekly incidence (median and IQRs) for both of the agent based models following treatment scale-up.

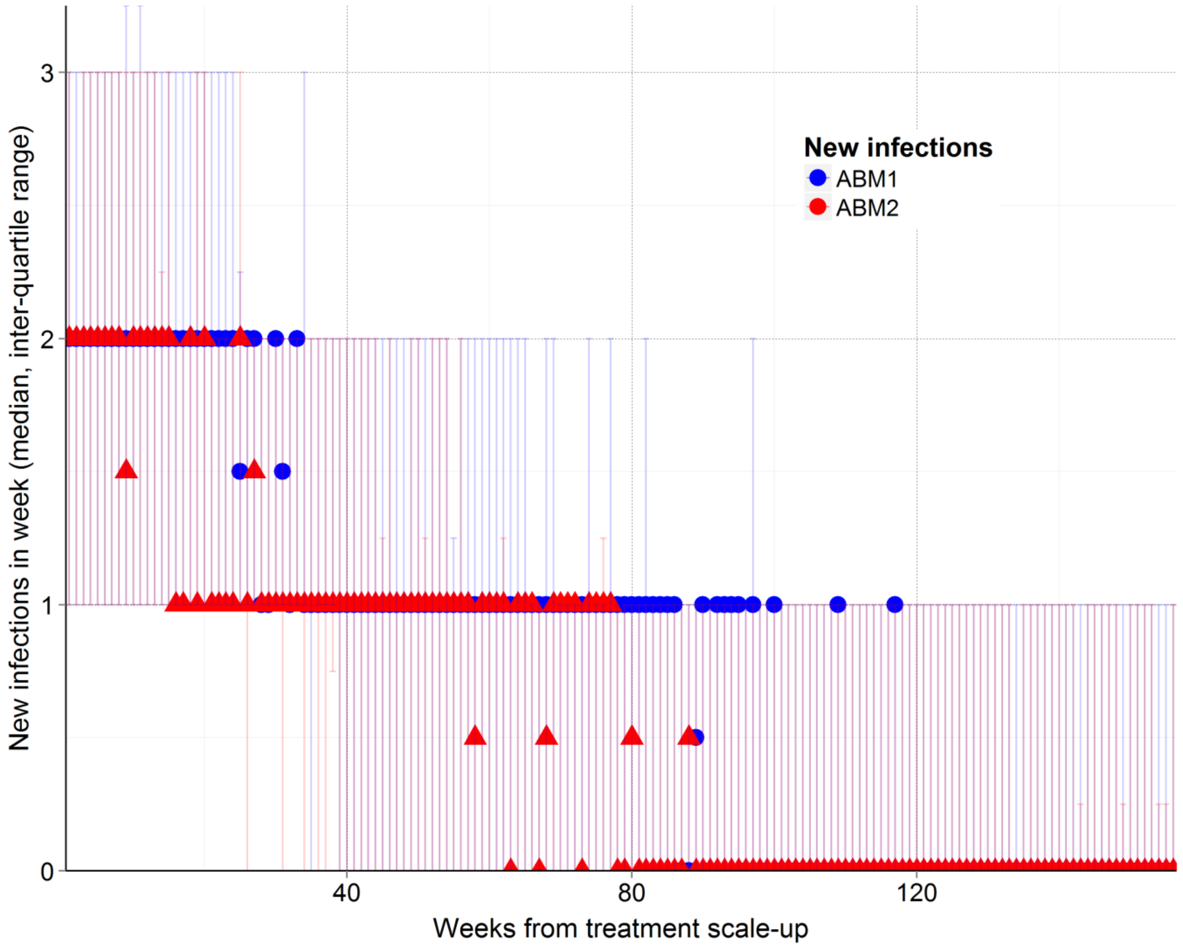

Figure S4: Projected incidence of HCV among HIV-positive MSM in Victoria over the first three years of treatment scale-up. The blue and red scatter plots represent median and inter-quartile ranges (IQRs) of the weekly incidence after multiple simulations for the first ABM (best estimates) and second ABM (more heterogeneous estimates) respectively.

Additional sensitivity and uncertainty analyses

Sensitivity analysis for the compartmental model

One-way sensitivity analyses were conducted to test the impact on compartmental model projections when: the additional relative risk of HCV infection and transmission for high-risk MSM was either 4 or 1 (i.e. no additional risk) compared to 2; the proportion of MSM defined to be at high risk was either 50% or 90% compared to 69%; the reduction in onwards transmission risk for MSM once they were diagnosed was either 0% or 90% compared to 45%; and the average time from diagnosis to treatment commencement was either 16 weeks or 52 weeks compared to 26 weeks.

The results of this analysis are shown in Table S1, compared to the baseline estimates. The greatest impact on outcomes was the average time from diagnosis to treatment commencement. Consistent

with the first ABM, significant reductions could be made in the time to reduce prevalence by 80% if this period was reduced to an average of 16 weeks, while if this time period were allowed to increase it would take considerably longer and require far more treatments to achieve the same prevalence reduction target.

Table S1: Sensitivity analysis for the compartmental model. Impact of alternate parameters on estimates of the time and treatment numbers required to reduce HCV prevalence among HIV-positive MSM by 80%.

	Time to reduce prevalence by 80%	Percentage difference from baseline estimate	Treatment numbers required to reduce prevalence by 80%	Percentage difference from baseline estimate
Baseline estimate	139		555	
Four times the relative risk for high-risk MSM (compared to double the risk)	136	-2%	551	-1%
No additional risk for high-risk MSM (compared to estimated double the risk)	146	+5%	564	+2%
50% of MSM at high risk (compared to estimated 69%)	143	+3%	559	+1%
90% of MSM at high risk (compared to estimated 69%)	137	-1%	553	-0(.3)%
No current risk reduction following diagnosis (compared to estimated 45%)	133	-4%	545	-2%
90% current risk reduction following diagnosis (compared to estimated 45%)	236	+70%	695	+25%
Average 16 weeks from diagnosis to treatment commencement (compared to 26 weeks)	102	-27%	524	-5%
Average 52 weeks from diagnosis to treatment commencement (compared to 26 weeks)	274	+97%	680	+22%

Latin Hypercube uncertainty analysis for the first ABM

In addition to understanding how individual parameter changes affect model outputs, Latin Hypercube Sampling [9-11] was used to test the effects of jointly varying model parameters. Continuous parameters were considered to be uniformly distributed between their lower and upper bounds, with 11 discrete sample points (10 intervals) used for each parameter. For example, the proportion of MSM with concurrent partners was tested at the values of 0%, 20%, 40%, 60%, 80% and 100%. This discrete decomposition of multiple parameters can be thought of as defining a hypercube lattice, with each lattice point representing a possible combination of parameters.

To attempt to separate variation due to parameter changes (i.e. variation across lattice points) from the stochastic variation of the model (i.e. random variability on each model run), 10 simulations were performed for each hypercube parameter sample and the average outputs were used as representatives of each lattice point. The distribution of average outputs from these 11^(number of parameters) hypercube sample points were compared to the baseline point estimate distribution with stochastic variation.

The large number of parameters made it unfeasible to perform this experiment on all variables at once, and so parameters were tested in three groups: 1) risk population-related parameters (proportion who have casual partners, proportion who have concurrent partners); 2) frequency-related parameters (average number of casual partners per person per year, average number of hook-ups per fuck buddy per year, percent of casual sex with partners outside of fuck buddy network); and 3) risk reduction-related parameters (condom use among casual partners, condom use among regular partners, risk reduction following HCV diagnosis).

The results are shown in Figure S5. Risk-reduction-related parameters had the greatest influence on model outcomes, consistent with the one-way sensitivity analysis (Figure 7). Relative to the stochastic variation of the model, variations due to parameter changes were modest, although it should be emphasised that each hypercube parameter sample used to generate the blue boxplots is associated with its own stochastic variation.

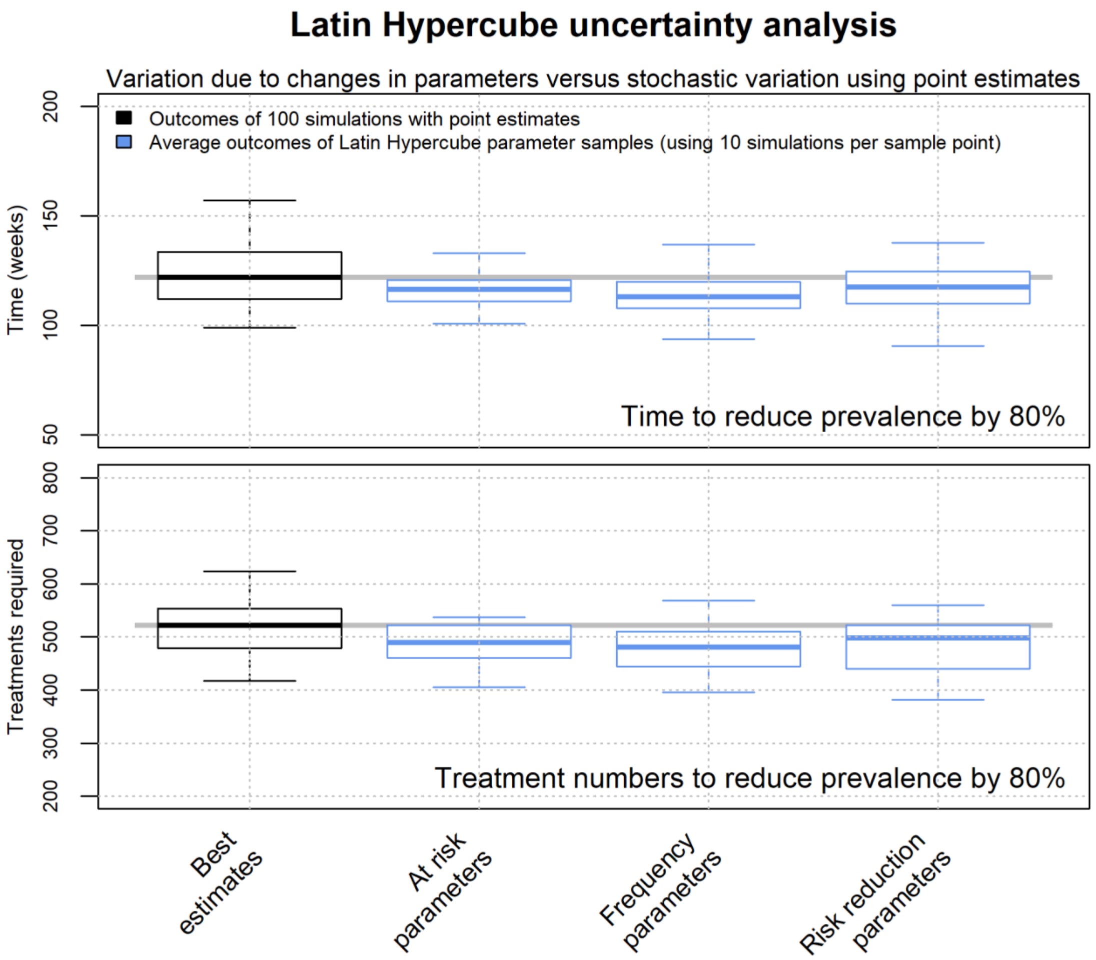

Figure S5: Latin Hypercube uncertainty analysis. Blue boxplots: Variation in the average (after 10 simulations) time and treatment numbers required to reduce HCV prevalence among HIV-positive MSM by 80%, as parameters move through points on the Latin Hypercube. Performed for risk population-related parameters (proportion who have casual partners, proportion who have concurrent partners), frequency-related parameters (average number of casual partners per year, average number of hook-ups per fuck buddy per year, percent of casual sex with partners outside of fuck buddy network), and risk reduction-related parameters (condom use among casual partners, condom use among regular partners, risk reduction following HCV diagnosis). Black boxplot: stochastic variation from 100 simulations with point estimate parameters.

References

1. Wilkinson A, El-Hayek C, Fairley C, Roth N, Tee B, McBryde E, Hellard M, Stoové M: **Measuring transitions in sexual risk among men who have sex with men: the novel use of latent class and latent transition analysis in HIV sentinel surveillance.** *American Journal of Epidemiology* 2016, **185**(8):627-635.
2. Cuevas M, Lim M, Nguyen P, El-Hayek C: **Hepatitis C Testing and Infection in HIV-Positive Men in Melbourne, Victoria. A Retrospective Cohort Study**. *Prepared by the Burnet Institute for the Victorian Department of Health* 2012.

3. El-Hayek C, Doyle J, Cuevas M, Lim M, Fairley C, Leslie D, Roth N, Tee B, Stoove M, Hellard M: **P733 New hepatitis C infection and re-infection among HIV co-infected men in Melbourne, Australia**. *Journal of Hepatology* 2014, **60**(1):S314.
4. Diekmann O, Heesterbeek J, Metz JA: **On the definition and the computation of the basic reproduction ratio R 0 in models for infectious diseases in heterogeneous populations**. *Journal of mathematical biology* 1990, **28**(4):365-382.
5. Van den Driessche P, Watmough J: **Reproduction numbers and sub-threshold endemic equilibria for compartmental models of disease transmission**. *Mathematical biosciences* 2002, **180**(1):29-48.
6. Anderson RM, May RM, Anderson B: **Infectious diseases of humans: dynamics and control**, vol. 28: Wiley Online Library; 1992.
7. The Kirby Institute: **HIV, viral hepatitis and sexually transmissible infections in Australia Annual Surveillance Report 2016**. *The Kirby Institute, UNSW Australia, Sydney NSW 2052* 2016.
8. Grebely J: **The HCV Care Cascade: Mapping the HCV clinical care pathway in Australia. Available from https://www.eiseverywhere.com/file_uploads/18749081103e56477b53a3edad240ecb_JasonGrebely.pdf**. 2014.
9. Helton JC, Davis FJ: **Latin hypercube sampling and the propagation of uncertainty in analyses of complex systems**. *Reliability Engineering & System Safety* 2003, **81**(1):23-69.
10. Iman RL: **Latin Hypercube Sampling**. In: *Encyclopedia of Quantitative Risk Analysis and Assessment.* edn.: John Wiley & Sons, Ltd; 2008.
11. Marino S, Hogue IB, Ray CJ, Kirschner DE: **A methodology for performing global uncertainty and sensitivity analysis in systems biology**. *Journal of theoretical biology* 2008, **254**(1):178-196.
